# The effect of educational intervention based on theory of planned behavior on mothers’ skills in sexual care of children

**DOI:** 10.1186/s12889-022-14162-0

**Published:** 2022-09-16

**Authors:** Ali Khani Jeihooni, Arman Moradi, Asiyeh Yari, Amin Kiyani, Pooyan Afzali Hasirini

**Affiliations:** 1 Nutrition Research Center, Department of Public Health, School of Health, Shiraz University of Medical Sciences, Shiraz, Iran; 2 Department of Public Health, School of Health, Fasa University of Medical Sciences, Fasa, Iran; 3 Department of Health Education and Health Promotion, School of Health, Hormozgan University of Medical Sciences, Bandar Abbas, Iran; 4 Department of Public Health, School of Health, Kermanshah University of Medical Sciences, Kermanshah, Iran

**Keywords:** Children, Intention, Theory of Planned Behavior, Sexual Care

## Abstract

**Background:**

The parent’s and especially the mothers’ skills play a major role in the the education of healthy sexual behaviors in children. This study investigates the effect of educational intervention based on the Theory of Planned Behavior (TPB) on mothers’ skills in the sexual care of children in Fasa city, Fars province, Iran in 2019.

**Methods:**

This study was a quasi-experimental intervention with a control group. 200 mothers of children aged 5 to 6 years in Fasa preschool were selected using a multi-stage random sampling method and were divided into two groups of intervention (100) and control (100). After providing a pre-test to both groups, only the experimental group received training on sexual care of children's abilities based on the Theory of Planned Behavior constructs. The educational intervention consisted of seven 55–60-minute sessions in which the presenter gave a presentation, asked and answered questions, and used posters, brochures, films, animations, and PowerPoints. Both groups completed the questionnaire three months following the intervention. A questionnaire and Theory of planned behavior constructs were used to collect information. The data was analyzed with SPSS22 software using paired t-tests, Chi-square tests, and independent t-tests, with a significance level of 0.05.

**Results:**

Before the intervention, there was no significant difference in the constructs of theTheory of planned behavior between the two groups (*p*>0.05), but after the intervention, knowledge scores from 8.33±2.97 to 20.67±2.84, attitude scores from 29.80±4.27 to 62.22±4.34, subjective norms from 20.12±4.55 to 42.28±4.20, perceived behavioral control from 20.24±4.36 to 42.88±4.52, behavioral intention from 3.24±1.60 to 7.44±1.59 and behavior from 2.98±1.13 to 8.14±1.08 in the intervention group (*p*< 0.001).

**Conclusion:**

This study showed TPB constructs’ Effectiveness in adopting the level of mothers’ skills in the sexual care of children. Hence, this model can act as a framework for designing and implementing educational interventions for the sexual care of children.

## Background

People must undergo extensive sexual training in order to acquire the necessary information and understanding about sexual matters, which shapes their ideas, attitudes, and values. This type of education emphasizes all aspects of sexuality, including biological, cultural, social, psychological, and religious aspects. It also relates to cognitive (knowledge, information), emotional (emotions, values, and attitudes), and behavioral (communication and decision-making) skills [1].. A person who has received sexual training is protected against sexual misconduct by balancing and managing his or her natural sexual urges as well as by advancing social, ethical, and cultural development [2].. Sexual education refers to teaching in schools or other relevant classes on sexual health, sexual activity, and its effects on sexual health. This type of teaching ought to be compatible with people's sexual preferences, age, and cultural beliefs [3, 4]..

Sexual training is one of the most difficult pieces of training in which a small mistake may lead to children's misconduct in the future [5]. Parents play an important role in children's sexual health, evolution and self-confidence [6]. Jaccard et al. [7] mentioned three causes for how parental relationships with their kids effect their future sexual lives. First, parents can impart to their kids sexual knowledge that is congruent with social norms. Second, parents accompany their kids throughout all of the developmental stages they go through, including puberty and the changes that come with it. Third, parents are their children's primary source of information since they constantly observe their conduct. The bond between parents serves as a barrier against a variety of sexual habits in children in the future [8].. Pre-puberty era is an appropriate time for parents to educate children on prevention behaviors from sexual risks to children [9]. The Preschool era (3-6 years of age) is significant because children's characteristics, attitudes, knowledge, and behaviors evolved in this period [10].

One of the curiosity issues in these ages is sexual issues. Hence, the importance of education in this period is more obvious [11]. However, antiquated customs and a false sense of humility prevent Iranian families and the educational system from accepting this significant duty [12].. False modesty and ignorance are the two obstacles that prohibit parents and children from developing healthy sexual skills in each other. Actually, parental requirements in this field are not specified and they still have no proper definition about sexual training [13]. In most studies, mothers’ Role in sexual training is more significant. Mothers’ knowledge, whose children are mostly with them, is insufficient or incorrect [14–16]. In Iranian officials or unofficial educational system, there is no arranged program for sexual training for children [17, 18]. Parents are somehow afraid of talking about sexual issues with their children and think that, children's awareness about sexual issues will cause their misconduct and parents’ false modesty prevents them from giving sexual information to their children [19]. Parents should train these issues based on two following assumptions:

1. As skilled teachers, parents have sufficient knowledge about sexual issues.
2. Deciding on sexual training to children is under the control of parents [20].

Everyone is responsible for educating and balancing sexual instincts; however, the family has more responsibilities due to its priority in training and taking care of children [21]. Family plays an important role in the sexual training of children [22], and their Role as the first sexual educators is confirmed, and presenting educational sexual issues to parents is essential for children [23, 24]. Recent studies on sex differences and the relationship between children and parents have shown that moms play the most significant role as sexual educators at home and are more likely than fathers to discuss sexual problems with their kids [25].. Therefore, increasing mothers’ ability in the sexual care of children and recognizing their weak points are essential.

One of the most important health education and promotion models used in sexual health training theory of planned behavior (TPB) presented by Ajzen and Fishbein in 1988 is based on social, psychological, and social-recognition patterns [26]. History comprises attitude, subjective norms, perceived behavioral control, intention and behavior [27]. The best predictor of doing a behavior in TPB is intention [28]. The intention is an essential factor for performing a behavior [28], and by increasing the intention of doing a behavior, the success of that behavior becomes more possible [28, 29]. In most of conditions, evaluating behavior is actively or ethically impossible. Therefore, specialists believe that behavioral intention can be useful in evaluating real behavior in an intervention [30, 31]. Some studies use TPB to predict and promote healthy sexual behaviors and fertility [32, 33]. Turchik and Gidycz [34] and Sacolo et al. [35] defined TPB as a useful model for preventing risky sexual behaviors. According to the importance of sexual care in children and the need to educate mothers in this field, the purpose of this study is to investigate the effect of educational intervention based on TPB on mothers’ skills in sexual care of children in Fasa, Fars province, Iran in 2019.

## Methods

### Sampling

The current study is a quasi-experimental study conducted on 200 mothers with preschool children aged 5 to 6 years living in Fasa, Fars province, Iran, in 2019. Among 18 preschools in Fasa, Four preschool centers in Fasa from 4 different geographical regions that had a similar culture and social conditions were chosen at random, and 200 mothers from these four preschools, Based on the files of children, were chosen and asked to take part in this research: control group (100) intervention group (100 ). Lack of education in psychology, educational sciences, and medical sciences, as well as a history of not taking child sexual education courses, were inclusion criteria for this study. Lack of enthusiasm for involvement and missing more than two teaching sessions were both grounds for exclusion.

Following pilot research on 40 qualifying participants in this investigation, sample volume was computed for 100 participants for each group using the sample volume formula and a correlation coefficient of *P*=0.05 and a 15% decline.

$$n={\left(\frac{\left({Z}_{1- {\alpha }\!\left/ \ {2}\right.}+{Z}_{1-\beta}\right)}{0.5\times \mathit{\ln}\left(\frac{1+r}{1-r}\right)}\right)}^2+3$$

### Educational intervention sessions

Participants were introduced to each other, and the study's goals were communicated to them by inviting them to a specific day in the childcare facility. Participants were also informed that their information would be kept private and signed a consent statement. After selecting experimental and control groups, both groups completed a pre-test questionnaire. Following the experimental group's pre-test results, an educational intervention was carried out based on the notion of planned behavior. The educational intervention consisted of seven 55-60 minute sessions in which the presenter gave a presentation, asked and answered questions, and used posters, brochures, films, animations, and PowerPoints. Once a week, these seminars were held in the Fasa Department of Education's salon. Two health education and promotion specialists and two clinical psychologists held every session for 25 participants (4 groups with 25 members). Education was done face to face in group discussions (about mental beliefs, positive and negative consequences of a behavior, facilitating factors of doing a behavior, motivation for following other people's behaviors, and subjective norms) and through visual learning by presenting educational pamphlets, films, and animations. A director led a discussion and information exchange to determine positive ideas and attitudes to indirectly implant positive motivation for individuals to learn and perform tasks and change or modify unfavorable attitudes. In addition, various ways for managing and having self-confidence in child sexual care were addressed. As efficient subjective norms, one session was held in the presence of fathers, preschool authorities, department of education officials, doctors, and health center officials. The experimental group participants were separated into ten groups of ten people each, and the Role of peers and friends was stressed. A Whatsapp group was set up for them to exchange information, and an educational SMS was given to them once a week (Table 1).

**Table 1:** Educational intervention sessions

Training session	Constructs	Educational content	Time/ strategies
First and second	Knowledge & Attitude	Introduction and Objectives, Sexual development, sexual education, teaching children private places and sex games, sexual abuse	55-60 minute Brainstorm about all possible outcomes
Third and Fourth	Perceived Behavioral Control & Subjective Norms	The effect of peers and family on sexual care in children. Discussion of facilitators of sexual care behavior, incentives, reduction of deterrents. Role play by mothers, psychological drama, discussion and questions and answers	55-60 minute By influencing control beliefs and perceived power & By influencing normative beliefs and motivation to comply
Fifth and Sixth	Behavioral Intention & Behavior	Behavioral intent was taught by influencing attitudes toward behavior and mental norms, followed by encouraging mothers to engage in behaviors and strategies for providing sexual care to children.	55-60 minute By influencing attitude toward the behavior and subjective norms & By influencing a behavioral intention, which is dependent on attitude toward the behavior and subjective norms
Seventh	Review of previous sessions	The important topics of all the sessions were reviewed and all the constructs of theTheory of planned behavior became the focus of the training. discussion about sexual development, sexual training, teaching private issues to children, sexual games, sexual abuse, preventing sexual abuse, sexual self-care skills, and improving children's self-esteem	55-60 minute Role playing, mental show, panel discussion

### Follow-up and Measuring tools

A follow-up session was held once a month to track participants’ actions. Participants were given an instructional pamphlet at the end. It should be mentioned that the control group got no educational sessions, and at the end of the intervention, an instructional booklet was supplied to the control group for ethical observations. 3 months after the intervention, both groups filled out the questionnaire. The tool used for gathering information was a questionnaire provided based on other similar studies [1, 2, 7, 10, 14, 22, 27, 32]. The first component of the questionnaire asked for demographic information such as age, number of children, mother and father’s work, parents’ educational level, child’s sex, and information sources used for sexual care of children. The second segment featured questions evaluating the constructs of the Theory of planned behavior. 25 questions about knowledge were asked, with answers of “Yes,” “No,” and “No idea.” In this case, the correct answer received a score of one, while the incorrect or no idea response received a score of zero, ranging from 0 to 25. A five-point Likert scale ranging from 1 (totally disagree) to 5 (absolutely agree) was used to assess attitude, subjective norms, and perceived behavioral control. 15 questions were posed to assess attitudes, such as “there is no necessity for training children on sexual abuse prevention because children will learn it during the growing process.” The lowest and highest scores were 15 and 75, respectively. Ten questions were posed to assess subjective norms, such as “preschool administrators believe that sexual care teaching for children is vital” (the minimum score was 10, and the maximum score was 50). Ten items were used to assess perceived behavioral control, such as “I expected my child to say no to anyone who wanted to touch his/her sexual organ” (minimum score was 10, and the maximum score was 50). Ten questions with yes or no answers were used to assess behavioral intention, such as “I have attention to take care of my child sexually”, the lowest score was 0, and the maximum score was 10. Ten questions with yes or no answers were used to assess behavior, such as “Sexual care for my child is part of my upbringing,””, the lowest score was 0, and the maximum score was 10.

Mothers were monitored by telephone each month to ensure they were not being trained from sources other than our educational intervention.

### Validity and reliability of the questionnaire

The item impact size of the used questionnaire was greater than 0.15, and the content validity ratio was greater than 0.79. 40 mothers reviewed a list of prepared items with a preschool kid with similar demographic, economic, and social features to the investigated participants to determine the tool's face validity. The ideas of 12 specialists (from the research team) in health education and promotion (*n*=10) and clinical psychologists (*n*=2) were used to determine content validity. According to Lawshe's table, items with CVR values greater than 0.56 for 12 participants were deemed acceptable and maintained for further study. The calculated values for the majority of the items in this study were more than 0.70. Cronbach's alpha calculated the total consistency of the research instrument to be 0.87. Knowledge consistency was 0.88, the attitude was 0.89, subjective norms were 0.87, perceived behavioral control was 0.84, and behavioral intention was 0.87. Because the derived Cronbach's alpha values for each tested construct were greater than 0.70, tool consistency was correctly evaluated and confirmed.

The data were analyzed using SPSS22 software using paired t-tests, Chi-square tests, and independent t-tests, with a significance level of 0.05.

## Results

In this study, 200 mothers having a preschool child were investigated. The independent sample t-test indicated no significant differences between Demographic variables in two groups. (Table 2).

**Table 2:** Demographic characteristics of participants

Variables	Experimental group *N*=100	Control group *N*=100	*P*-value
	Frequency (n) Percentage (%)	Frequency (n) Percentage (%)	
Educational level of mother			
Illiterate	2	1	0.247
Elementary	12	13	
Middle school	35	32	
High school	39	41	
University	12	13	
Educational level of father			
Illiterate	1	0	0.186
Elementary	8	11	
Middle school	32	30	
High school	42	45	
University	18	14	
Mother's job			
Employed	19	16	0.314
Housewife	81	84	
Father's job			
Employed	78	73	0.285
Unemployed	22	27	
Child's sex			
Female	58	60	0.338
Male	42	40	

The results of the present study showed that the most important sources of information about child sexual care in both groups were books, physicians and health workers, the Internet, and teacher/school counselor. The mentioned cases 37%,34%, 17%,9% and 3% were in the intervention group and 34%,30%,21%,8%, and 7% were in the control group, respectively.

Results of this investigation showed that, based on an independent t-test, there was no significant difference between mean scores of constructs between experimental and control groups before the educational intervention. However, 3 months after the intervention, significant differences were observed in the experimental group. Also, the independent t-test showed that constructs’ mean and standard deviation score was significantly enhanced in the experimental group, while the control group had no changes (Table 3).

**Table 3:** Cross-comparison of research groups in TPB constructs pre-and post-test scores

Variables	Group	Pre-test Mean±SD	Post-test Mean±SD	Paired-sample T-test
Knowledge	Intervention	8.33±2.97	20.67±2.84	0.001
	Control	7.98±2.44	8.45±2.58	0.183
	Independent-sample T-test	0.166	0.001	
Perceived Behavioral Control	Intervention	20.24±4.36	42.88±4.52	0.001
	Control	21.64±4.39	23.12±4.14	0.171
	Independent-sample T-test	0.149	0.001	
Subjective Norms	Intervention	20.12±4.55	42.28±4.20	0.001
	Control	20.88±4.62	21.89±4.41	0.175
	Independent-sample T-test	0.198	0.001	
Attitude	Intervention	29.80±4.27	62.22±4.34	0.001
	Control	28.95±4.14	29.72±4.83	0.186
	Independent-sample T-test	0.212	0.001	
Behavioral Intention	Intervention	3.24±1.60	7.44±1.59	0.001
	Control	3.81±1.55	4.02±1.52	0.233
	Independent-sample T-test	0.192	0.001	
Behavior	Intervention	2.98±1.13	8.14±1.08	0.001
	Control	3.06±1.16	3.34±1.15	0.258
	Independent-sample T-test	0.288	0.001	

## Discussion

As with other existential dimensions of human beings, sexual instinct needs pedagogy. Hence, knowledge, attitude, and parents’ skills, especially mothers, play the most important Role in training the sexual behaviors of children in the preschool era [36]. As a child, children discover their sexual organs and their sexual instinct becomes activated, which should be accompanied by the observation and control of parents because premature sexual behaviors can culminate in irrecoverable harm. By training sexual care for children at an appropriate age, parents can gradually immune their children from sexual abuse [37]. The present study investigates the effect of educational intervention based on the Theory of planned behavior on mothers’ skills in sexual care of preschool children in Fasa, Iran, in 2019.

Before the educational intervention, the score of mothers’ knowledge of sexual care of their children in the two studied groups was low; however, 3 months after educational intervention, the mean score of knowledge of the experimental group showed significant enhancement compared to the control group. The most important information sources in studied participants about sexual care of children were, respectively, books, doctors, health officials, the Internet, and teacher or consultant. In a study by Rashid and Hosseini Nazarbu [38], holding seven educational sessions caused an increase in sexual knowledge and the responsibility of parents about the sexual training of their children. Parents’ knowledge about sexual issues, such as understanding natural and unnatural sexual behaviors, treating children suffering from sexual abuse, the time of educating sexual issues, forming the sexual identity of the child, puberty, relationship with peers, information sources of children, etc. are effective factors in preventing inappropriate behaviors in children and improving self-efficacy of parents [39]. In the study by Mobredi et al. [40], mothers’ attitudes and knowledge about sexual training in preschool children were mean, and a significant relationship was found between mothers’ attitude and their educational level. In studies by Aral et al. [41] and Kurtuncu et al. [10], participants believed that sexual training should be started in the preschool era. Haruna et al. [42] used sexual health education program for adult students through game-based learning and indicated that, after educational intervention, significant statistical differences were observed in the knowledge and attitude of participants. Margo Rule et al. [43] investigated effective factors for mandatory reporting of sexual abuse in children by primary school teachers in South Africa based on TPB and found that almost 25% of teachers had reported at least one case of sexual abuse in children during their teaching career and 7% of them had failed to report the suspected sexual abuse case. According to the results of the present study, it can be concluded that the current education has a greater effect on raising the level of mothers’ awareness than non-systematic education and therefore, it is necessary to train children in sex care centers.

In the current study, 3 months after educational intervention, significant enhancement was observed in the mean attitude score in the experimental group. Also, a positive attitude caused an increase in mothers’ skills in the sexual care of children. Presenting educational films, animations, group discussions, and presenting beliefs caused the creation of positive attitudes toward learning and performing skills and modifying negative attitudes of mothers. Also, sending educational and motivational SMS helped the promotion of participants’ attitudes. In a study by Moeini et al. [44], educational intervention based on the Theory of planned behavior caused an increase in participants’ attitudes toward healthy sexual behaviors in the experimental group. In Bayley et al. [45] study based on the Theory of planned behavior, attitude predicted the intention. Results of the present study are in good agreement with the results of Sanberk et al. [46], who investigated the attitude of Turkish mothers having a child with the age of 48-66 months and revealed that educational intervention caused the increase in participants’ attitude, and also with the study of Khani Jeihooni et al. [47] who studied prevention behaviors from sexually transmitted diseases. Nagpal et al. [3] indicated that important changes had been created in parents’ attitudes toward sexual training in children, and in recent years, parents’ attitudes have changed from negative to positive. Investigating sexual behaviors in developing countries was always influenced by different and conflicting attitudes. Due to cultural limitations, sexual training is not performed in schools, and discussing sexual issues with children is unnatural for parents [3]. Forouzi et al. [48] indicated that most parents have negative attitudes toward sexual training in teenagers and suggested changing attitudes and increasing parents’ knowledge in this field. Kalantari et al. [49] studied mothers’ experiences in training puberty and the sexual behaviors of their daughters. They concluded that, unlike recent cultural changes in Iran, sexual training is insufficient due to the dominant common culture in society and families about sexual puberty.

The mean score of perceived behavioral control had significant enhancement in the experimental group 3 months after the intervention, while the control group had no changes. Perceived behavioral control is an individual's beliefs about the availability or unavailability of sources and chances for doing a specific behavior, and when encountered with challenges, the individual feels that he/she can control that behavior [50]. In this study, presenting educational films and images, training sexual care by psychologists, interesting mothers in learning skills, increasing their self-confidence, providing Whatsapp groups for exchanging information, and sending educational and motivational SMS caused the increase in the mean score of perceived behavioral control in the experimental group. Preventing sexual abuse in children is the adults’ responsibility. The best prevention and protection method is adults’ consciousness who never abandon their children in difficult conditions, understand their discomfort, and listen to their words. Therefore, families should educate life principles and patterns in social relationships because family is one of the most efficient structural patterns in training skills, such as having appropriate behavior in dangerous conditions, including sexual abuse [51, 52]. In the study of Jalam badani et al. [53], the experimental group's mean scores of knowledge, attitude, perceived behavioral control and intention of sexual function were significantly increased after the educational intervention.

In the current study, 3 months after the intervention, the mean score of subjective norms of the experimental group increased, while the control group had no changes. Because subjective norms are affected by important people in an individual's life and also because of holding educational sessions for fathers, doctors, health officials, and preschool officials, subjective norms of the experimental group are enhanced. Also, the studied participants were divided into 10 groups with 10 members (friends and peers groups), which caused an increase of knowledge and positive attitude of participants and learning skills. In study of Sarayloo et al. [54], educational intervention caused an increase of knowledge, attitude and subjective norms of experimental group. A quasi-experimental study of Mousali et al. [44] indicated that, educational intervention based on TPB causes the increase of the mean score of subjective norms in the experimental group. In study of Eggers et al. [55], subjective norms predicted healthy sexual behaviors. In a study of Khouii et al. [56] entitled “Sexual training in Iranian students by health educators in elementary schools”, it was revealed that most parents believed that sexual evolution of children should happen in the family environment. Family-based sexual education is one of the topics that can help parents to be effective in the sexual behaviors of their children [22]. This training improves parents’ knowledge, attitude and performance in sexual training in children [57]. In the study of Hemat et al. [58] about maternal attitudes toward child sexual abuse, there was significant differences in mean scores of attitude and subjective norms of experimental and control groups after intervention. Results of Kurtuncu et al. [10] showed that, the number of correct responses given by participants regarding some behaviors of children with the age of 3-6 years and their sexual development showed significant differences in age, marital status, number of children and educational and job status of parents. It was observed that culture has an important effect on sex-related approaches and embarrassment and shyness are very common factors among people. In studies of Wilson et al. [59] and Knabe et al. [60], subjective norms predicted the intention in the experimental group.

In the study of Larki et al. [61], educational intervention based on TPB was performed in 7 sessions and after that, significant enhancement was observed in the mean score of perceived behavioral control, attitude and subjective norms of the experimental group about risky sexual behaviors. Wong et al. [62] investigated a behavioral intervention for promoting the use of condoms in prostitute women. In his study, educational intervention caused an increase of self-efficacy of the experimental group compared to the control group in terms of discussion skills and rejecting risky sexual offers. In the quasi-experimental study of Ebrahipoor et al. [63], after educational intervention based on TPB, knowledge, attitude and perceived behavioral control of the studied subjects increased and the sexual performance of the experimental group had significant enhancement. According to the definition of perceived behavioral control, which indicates barriers and facilities of doing a behavior [64] and the traditional culture of Fasa, which does not accept such behaviors, perceived behavioral control is highly important. In the current study, the mean score of perceived behavioral control showed the effect of education on removing barriers to behavior. Increasing the variables of knowledge, attitude, behavioral control, perception and abstract norms towards child sexual care are important factors that led to the promotion of intention and as a result, sexual skills of mothers and children in this study. When mothers have sufficient and correct knowledge with a positive attitude towards child sexual care and can sexually care for children, environmental factors are also at their disposal and on the other hand, contemporary abstract norms such as spouse, doctor and health workers. The therapists and education officials and preschools encourage them, and then their skills increase.

Current research results revealed that, the mean score of participants’ intention for sexual care of children and their behavior had significant enhancement 3 months after the intervention, while control group had no changes. Increasing an individual's intention for doing a specific behavior enhances the possibility of doing that behavior [65]. The increase of knowledge, attitude, perceived behavioral control and subjective norms in sexual care of children are important factors caused the promotion of participants’ intention and sexual care skills of mothers. When mothers have proper and sufficient knowledge with a positive attitude toward sexual care of children, their ability in sexual care of their children enhances and environmental factors become under their control. On the other hand, subjective norms such as fathers, doctors, health officials and education officials encourage them and mothers’ training skills enhance.

Cha et al. [66] investigated the constructs of attitude, perceived behavioral control, subjective norms and participants’ intention for premarital sex and found that, perceived behavioral control do not predict the intention for premarital sex in studied participants. In the study of Moeini et al. [44], educational intervention based on the Theory of planned behavior caused an increase of behavioral intention and promotion of healthy sexual behaviors in the experimental group. Hashemi Bakhshi et al. [67] investigated the effect of theory-based educational intervention on mothers’ skills in sexual care of elementary school students. In his study, educational intervention caused significant enhancement in mean scores of attitude, subjective norms, behavioral intention and behavior in the experimental group, while the control group had no changes. In study of Khanjari et al. [68] to determine the effect of education on preventing child sexual abuse and parents’ performance by giving the presentation, asking and answering questions, presenting films, etc. and 6 months after the intervention, the mean score of experimental group’s performance significantly enhanced. In a study of Chen et al. [51], who investigated parents’ knowledge, attitude and performance in educating child sexual abuse prevention, it was revealed that 60% of parents had told their children that other people should not touch their sexual organs. On the other hand, only 4.2% of parents had provided books or other training tools about sexual abuse prevention for children and according to mentioned study, knowledge and performance of parents in preventing child sexual abuse were insufficient.

The leerlooijer et al. study showed significant associations to obtain from sexual intercourse were found for experience with sexual intercourse, perceived behavioral control, attitude and subjective norms of peers and parents, explaining 31% of the variance in abstinence intention [69].

Lynn Miller et al. [70] used TPB to the proximal and distal etiology of sexual offending. In study of Margo Rule et al. [43], subjective norms and perceived behavioral control predicted the intention of teachers to report child sexual abuse. A teacher who reported child sexual abuse in the past as well as a teacher with more accurate knowledge in mandatory reporting were more likely to have the intention to report such issues in future. These findings probably indicate the impact of the designed training program.

One of the limitations of this study was the self-reporting answers of participants. The other limitation was lack of appropriate cooperation of mothers due to cultural limitations and modesty in sexual training. Of course, by giving demanded information, authors tried to eliminate these limitations.

## Conclusion

Results of the present study revealed that educational intervention based on the Theory of planned behavior greatly affects a mother's skills in sexual care of children, their attitude, subjective norms, perceived behavioral control, behavioral intention, and behavior. Educating sexual issues should be performed based on organized programs in schools, and educational officials, such as the department of education, school managers, teachers, and preschool officials should be educated in this field. In order to design educational and social interventions for preventing risky behaviors in children, teenagers, and youths, educational theories and models should be used.

## Data Availability

The datasets generated during and analyzed during the current study are publicly available from the corresponding author request.

## Abbreviation

TPB: Theory of Planned Behavior
